# Design and Realization of Wearable Textile Slotted Waveguide Antennas

**DOI:** 10.3390/s23177509

**Published:** 2023-08-29

**Authors:** Davorin Mikulić, Evita Šopp, Davor Bonefačić, Juraj Bartolić, Zvonimir Šipuš

**Affiliations:** Faculty of Electrical Engineering and Computing, University of Zagreb, 10000 Zagreb, Croatia; davorin.mikulic@fer.hr (D.M.); evita.sopp@fer.hr (E.Š.); davor.bonefacic@fer.hr (D.B.); juraj.bartolic@fer.hr (J.B.)

**Keywords:** wearable antennas, textile antennas, slotted waveguide antennas, waveguide to coax transitions, conductive textile

## Abstract

The design of wearable antennas presents a challenge from multiple perspectives, as they must meet technical requirements and satisfy safety standards while also being suitable for integration into clothing and aesthetically pleasing. In recent years, the development of conductive fabrics has, in many ways, allowed for significant progress in the manufacturing of wearable antennas, and in previous work, we developed textile slotted waveguide antennas using conductive textiles and traditional sewing processes. However, various aspects of the design and realization of such antennas remain challenging. In particular, this work investigates the issue of using foam-based molds, which enables the realization of thin, flexible, wearable antennas, as well as the issue of antenna feed, specifically the transition from a classic coaxial transmission line to a waveguide. The design of the transition was focused on simplicity and robustness, due to which we limited the number of degrees of freedom in the design process in order to achieve a structure suitable for mounting on textile waveguide antennas. In addition, the antenna design procedure and the body-channel model were considered in order to optimize the performance of the antennas and the wireless body-centric system itself. Several prototypes of different kinds were developed in the 5.8 GHz ISM band, confirming the feasibility of the proposed concepts through experimental results.

## 1. Introduction

**Figure 1:**
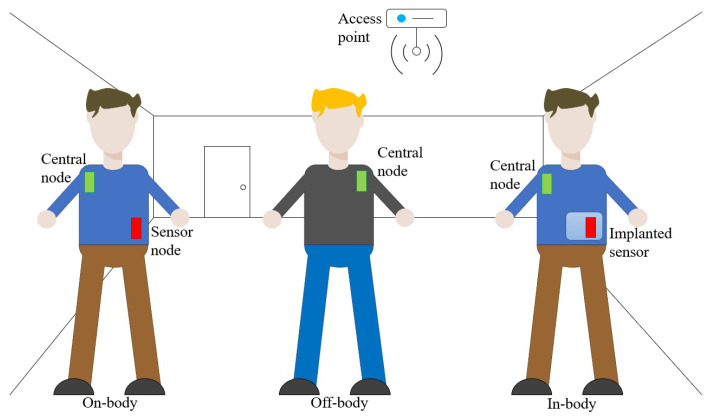
Different scenarios of body-centric wireless sensor and communication systems.

In recent years, there has been an increased interest in body-centric sensor and communication systems due to their wide range of applications, which aim to enhance quality of life by improving sensing, detection, and monitoring capabilities [1,2,3]. To meet the requirements of these applications, wearable antennas must be portable, flexible, and capable of integrating into clothing or uniforms. As a result, these antennas must simultaneously meet technical requirements, safety regulations, aesthetic standards, and the demands of wearable applications. In general, body-centric wireless systems are divided into three groups: off-body, on-body, and in-body systems, each with its own design challenges [4]. The on-body and off-body modes of operation are mainly related to the communication of different wearable sensors with a central node to aggregate measured data and transmit them to the access point (see Figure 1). In the in-body mode of operation, in addition to a reliable communication link between implanted and external monitoring devices, wireless power transfer (WPT) systems are often required to solve the problem of powering the implanted devices. A wide range of applications has been proposed, ranging from the ability to monitor bio signals and bio parameters to the capacity to interact with the environment. This continuous stream of information about a person’s health status can provide valuable medical insights, in addition to enabling the tracking of fitness levels in healthy individuals. Such systems can also be applied in emergency operations, such as firefighting operations. Communication capabilities can find use in contexts such as the implementation of safety vest systems necessary in environments where human–robot collaboration is essential, such as in automated warehouses [5].

From the user’s perspective, factors like flexibility and comfort are of concern. Therefore, antennas should be seamlessly integrated into garments. Two approaches exist for creating such antennas: one involves using non-textile materials, like flexible copper antennas, customized ornamental antennas, or plaster-like antennas designed for direct application on the skin, and the alternative approach is to employ textile antennas [6]. Since the antennas under consideration are mostly integrated into clothing, designs made of conductive textiles are preferred, as they allow the wearer freedom in daily activities while enabling connectivity between devices.

The vast majority of existing wearable antennas are dipoles, monopoles, planar inverted Fs (PIFAs), microstrip patches, and planar low-profile antennas in general [2,7,8,9,10]. However, other types of antennas can also be adapted to be suitable for wearable applications. The aim of this work is to further investigate the properties of wearable realization of a slotted waveguide antenna [11] made using conductive fabric. A waveguide slot antenna made of a textile-filled waveguide is presented in [12], but the waveguide walls are made of copper foil, which is impractical for wearable applications. The application of surface-integrated waveguides (SIWs), adapted for textiles as textile-integrated waveguides (TIWs) [13,14,15,16,17], has also been proposed. A TIW comprises three layers: the top and bottom conductive layers are typically constructed from conductive fabric, while the central dielectric layer typically consists of traditional materials such as wool felt or polyester-based textile. The creation of the narrow waveguide walls is adapted to textile technology, often achieved using conductive thread or eyelets. TIWs are well-suited for higher frequencies, e.g., in the millimeter-wave frequency range. However, for lower frequencies, it is necessary to employ multiple layers of textile, potentially resulting in a bulkier design. Moreover, due to the utilization of conductive thread or eyelets in achieving narrow waveguide walls, incorporating slots onto them becomes unfeasible. This limitation becomes especially pronounced when developing waveguide antennas for on-body modes of operation (as discussed in detail in Section 4).

In previous work [18,19], we proposed a waveguide antenna that was fully realized using conductive fabric and a sewing manufacturing procedure, which is a natural way of making textile objects. This allows for easy installation of the proposed antenna into a jacket, smart vest, belt, or other clothing item. We theoretically demonstrated the robustness of the proposed solution in all three (off-body, on-body, and in-body) communication scenarios. The experimental realization was focused on the radiation properties and inherent losses of the textile slotted waveguide array; therefore, we fed the developed antennas using a commercial waveguide-to-coax adapter, which should be replaced in the final design. Furthermore, the material used for mold realization was ridged and quite thick; thus, improvements are possible in order to achieve a fully wearable and bendable solution. Therefore, the goal of this paper is to discuss different aspects of the realization of textile slotted waveguide antennas, including the design procedure, suitable feeding structure (coax-to-waveguide transitions with emphasis on simplicity of design), mold selection to enable the creation of a thin and flexible antenna structure, etc. Various feeding structures were initially considered in [20]. However, this paper is focused on presenting a systematic design approach and discussing the antennas that were subsequently realized based on this approach in detail.

This paper is organized as follows. Section 2 presents the realization and basic properties of textile slotted waveguide antennas with an emphasis on different modes of operation: off-body, on-body, and in-body scenarios. The problem of the feeding structure is discussed in Section 3, and three types of coax-to-waveguide transitions are proposed and characterized. Six textile antenna prototypes are manufactured and experimentally characterized, and a comparison of measured and calculated results is presented in Section 4. An approach to estimate the coupling level between wearable antennas in the on-body mode of operation is also discussed. Finally, conclusions are presented in Section 5.

## 2. Realization of Textile Slotted Waveguide Antenna

Body-centric wireless communication systems have many potential applications. Antennas used in such systems are typically classified into three scenarios based on the communication channel: on-body, off-body, and in-body mode of operation. Figure 1 illustrates the typical applications for each scenario. In on-body communication systems, antennas are designed to enable the propagation of electromagnetic waves along the surface of the human body, allowing for seamless communication between sensors and central nodes (gateways). Conversely, off-body antennas require a wide beam around the human body to establish communication between a central node on the human body and access points positioned at arbitrary locations. Lastly, in-body antennas are optimized to efficiently radiate electromagnetic waves into the human body, enabling reliable communication between central nodes and implantable sensors, as well as facilitating power transfer to these devices. Therefore, the actual realization of a slotted waveguide antenna depends on the specific application.

**Figure 2:**
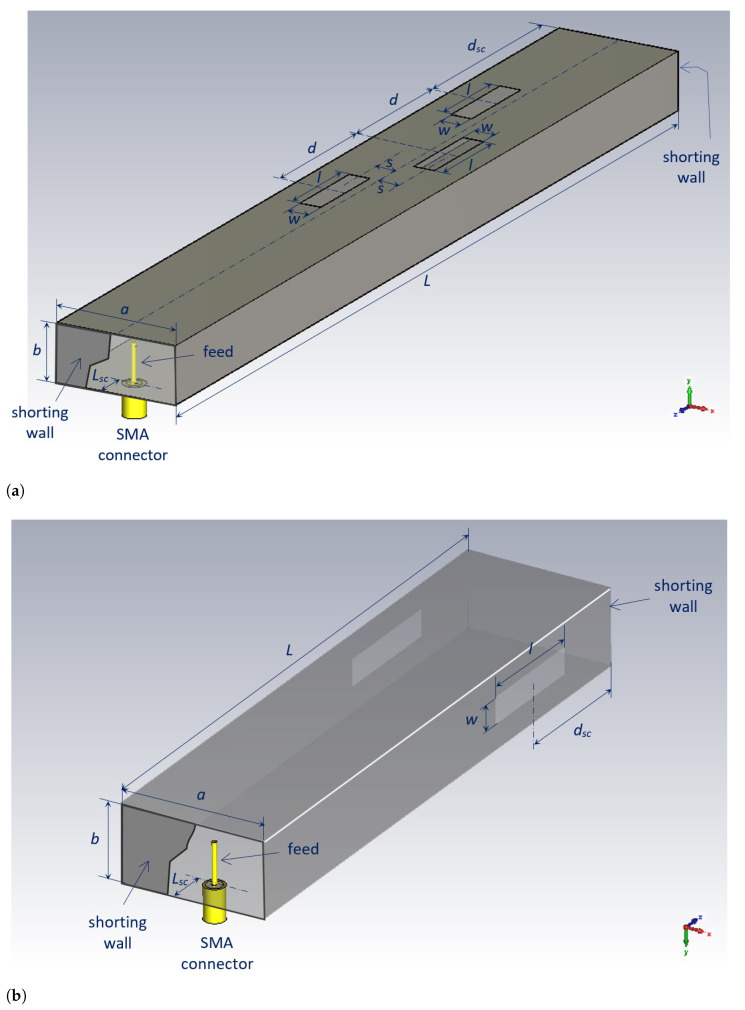
Schematic representation of the three-element slotted waveguide array for the off-body mode of operation (**a**) and of the waveguide array with a pair of slots in the narrow waveguide walls for the on-body mode of operation (**b**).

As examples, two different antenna designs are presented in Figure 2 for off-body and on-body types of communication. In the first case, the array contains three slots that are half the guided wavelength apart and approximately half a wavelength long. The waveguide is short-circuited at a distance of three-quarters of the guided wavelength from the center of the third slot. By doing so, each slot radiates a portion of both a forward and backward propagating wave, resulting in increased radiated EM power per slot. Note that most of the energy is radiated out from the body; thus, the regulations concerning the body specific absorption rate (SAR) values are easily satisfied.

For the on-body mode of operation, the slots are cut into the narrow waveguide walls, resulting in the launch of the normally polarized EM wave along the body. Additionally, the penetration depth of EM fields into the human body is very small, fulfilling the safety requirements. For both cases, the calculated SAR values and the corresponding maximum input antenna powers are given in [18].

In order to investigate the properties of the proposed textile slotted waveguide antenna, we designed and experimentally verified several textile waveguide antennas operating in the 5.8 GHz ISM band. A schematic representation of the considered textile antennas is shown in Figure 2. In principle, the antenna is made of a piece of waveguide on which slots are cut out and which is short-circuited at the end. The waveguide walls were made of conductive textile, and all connections of the walls were realized by a classical sewing procedure. The radiating slots were cut out, and the borders were sewn to fix the dimensions and prevent tearing. The sewn slotted waveguide, with a shape resembling that of a sock, was pulled over a mold (i.e., over an appropriate supporting structure) in order to keep the desired cross section of the antenna.

For the conductive fabric, we selected Shieldex®Nora Dell No.: 1401101S80 [21]. This conductive textile is made of Ni/Cu/Ag-plated polyamide fabric with an average surface resistivity of 0.009 Ω/□ and a thickness of 0.125 mm ± 15%. To create a mold, two materials were considered: rigid molds made from styrodur planar sheets with a permittivity of $\varepsilon_{r}\approx 1.02$ [18] and bendable molds made using Cuming Microwave C-FOAM PF-4 sheets with a permittivity of $\varepsilon_{r}\approx 1.06$ [22]. The use of a bendable mold offers several advantages compared to the use of a stiff Styrofoam mold. The bendable mold is thinner, making it more suitable for integration into clothing and enabling the realization of flexible textile waveguide antennas. Therefore, it is important to further investigate and experimentally characterize the performance of textile waveguide antennas with a thin foam-based mold. This will help to determine the feasibility and potential benefits of using a thin bendable mold for antenna integration into clothing and wearable applications.

## 3. Feeding Possibilities

The design of the considered waveguide antennas is divided into two parts, where the dimensions are separately optimized. First, the transition from an SMA coaxial connector to a rectangular waveguide is designed. Then, in the second step, a slotted waveguide array is constructed. In the first step, the waveguide is loaded with the matched waveguide port, ensuring that all the power entering the waveguide is transmitted to the waveguide port. In the second step, the antenna is excited with a waveguide-dominant mode using a waveguide port in a general electromagnetic solver (CST in our case [23]). In the final step, the two designed structures are joined together, and the antenna dimensions are fine-tuned. One could also design the feeding structure and the antenna part together. However, in such a case, there is a non-negligible possibility of designing the antenna as a resonator loaded with radiating slots (note that the considered waveguide section is short-circuited at both sides; see Figure 2), leading to narrow operating bandwidth design.

**Figure 3:**
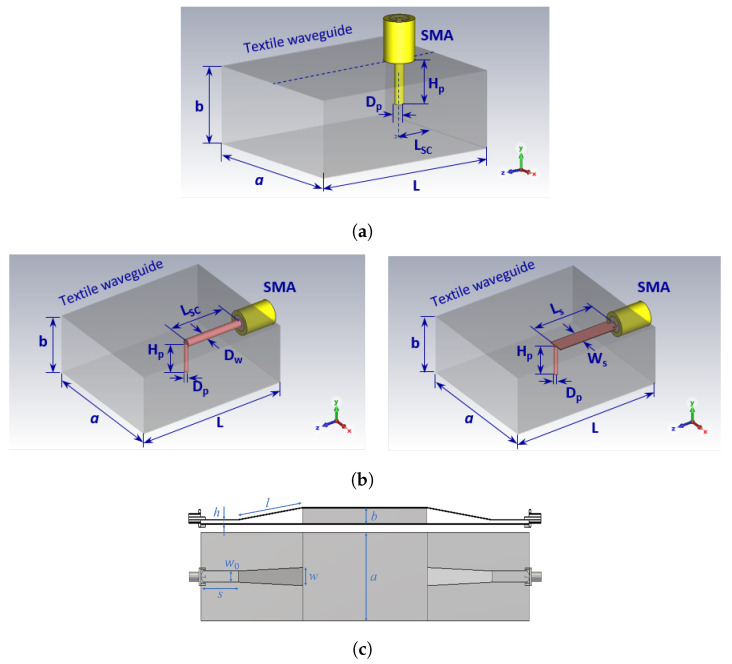
Schematic representation of waveguide feeding structures: (**a**) top- or bottom-mounted coaxial probe (transition A); (**b**) edge-mounted coaxial probe (transition B); (**c**) microstrip line to waveguide (transition C).

In this section, we discuss three different types of feeding transitions, as shown in Figure 3:

- Transition A: Top- or bottom-mounted coax-to-waveguide transition;
- Transition B: Edge-mounted coax-to-waveguide transition;
- Transition C: Microstrip line-to-waveguide transition.

The width and height of the waveguide are denoted as *a* and *b*, respectively, while the permittivity of the waveguide filling is denoted as $\varepsilon_{r}$. Two types of molds were considered: a rigid mold with a height of b=15.8 mm (made from Styrodur) and a bendable mold with a height of b=6.35 mm (made from foam PF-4). Final waveguide dimensions are provided in Table 1. Transitions A and C were later experimentally realized and integrated with the antenna to characterize their properties and their influence on the radiation properties of the antenna.

**Figure 4:**
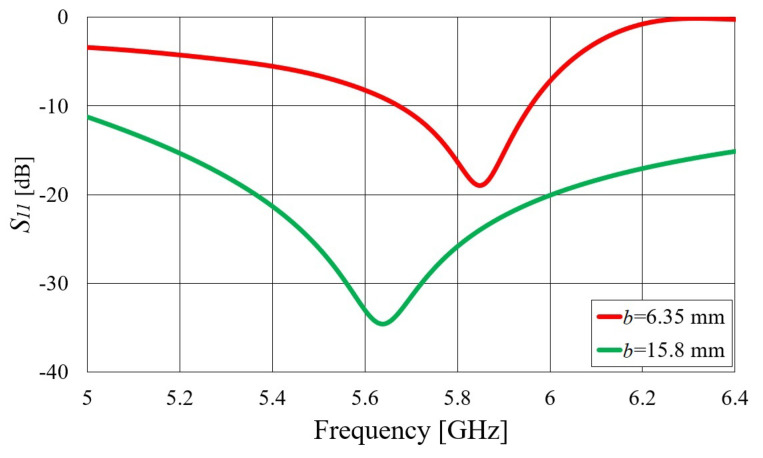
Simulated magnitude of the $S_{11}$ parameter of transition A (calculated at the SMA port) for two waveguide realizations with different waveguide heights (*b*). The considered structure is illustrated in Figure 3a.

Transitions A and B were designed using a bottom- and edge-mounted SMA connector, respectively (Radiall R125.414.000 was used in all prototypes), creating a structure similar to that of a waveguide-to-coax adapter. However, unlike high-quality adapters, our focus was on simplicity of design, i.e., the goal was to design a “good enough” transition covering at least the 5.8 GHz ISM band (5.725–5.875 GHz). Therefore, related to transition A, only two parameters were varied to obtain an optimal design: the length of the pin (the diameter was equal to the diameter of the inner conductor of the coaxial connector, i.e., 1.28 mm) and the distance from the short circuit. In the case of a thinner waveguide (*b* = 6.35 mm), we decided to extend the pin to the opposite side of the waveguide wall and solder it to that textile wall, obtaining good contact. Thus, in the design of a thin waveguide, only one dimension was available for optimization: the distance from the short circuit. Since the capacitive part of the matching circuit cannot be obtained by changing the length of the pin, the only way to add the capacitive part is to place the feeding point quite far away (slightly less than $\lambda_{g}/2$) from the short-circuit wall where the shorted waveguide section shows a capacitive reactance. Therefore, this distance is now much larger—30.5 mm versus 9.0 mm as in the previous case (all the dimensions are given in Table 2). One should note that a larger bandwidth can be obtained by employing a shorter pin (like in the case of a thicker waveguide). However, such a design is more sensitive to parameter variation. In Figure 4, the magnitude of the $S_{11}$ parameter is given for both designs. Although the considered structure is very simple, with only one or two degrees of freedom, good impedance matching in the entire 5.8 GHz ISM band is achieved. Of course, allowing additional degrees of freedom for optimization would result in better impedance matching in a larger bandwidth.

Transition B consists of an edge-mounted SMA connector feeding a shorting elbow. There are two possibilities with respect to how to feed the shorting elbow: with a wire or with a strip. In contrast to transition A, which essentially excites the waveguide with an *E* field from a monopole, transition B produces excitation through an *H* field with a current loop. However, transition B is more complicated than transition A, as it has four degrees of freedom. Due to this complexity, transition B is not experimentally realized or integrated with textile waveguide antennas. Nonetheless, the optimized dimensions for transition B, including both wire and strip-fed shorting elbows, as well as for rigid and bendable molds, are provided in Table 3.

**Figure 5:**
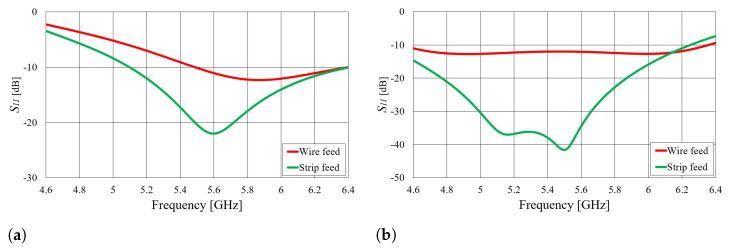
Simulated magnitude of the $S_{11}$ parameter of transition B (calculated at the SMA port) for two waveguide realizations with different waveguide heights (*b*): (**a**) *b* = 15.8 mm and (**b**) *b* = 6.35 mm. The considered structure is illustrated in Figure 3b.

Figure 5 shows the calculated $S_{11}$ parameters of transitions B for both types of molds. As shown in Figure 5, both wire-fed and strip-fed transitions B provide matching in the 5.8 GHz ISM band. In general, a strip-fed shorting elbow tends to provide better impedance matching compared to a wire-fed shorting elbow. This is because the strip provides a larger surface area for the EM wave to couple into the waveguide, resulting in improved power transfer and reduced reflection.

The final considered transition is a microstrip line-to-rectangular waveguide transition, as shown in Figure 3c. The transition includes an SMA connector, a 50 Ω microstrip transmission line, and a linear microstrip taper. Since in this case, radiation losses are present, a symmetrical two-port cascade network of the waveguide and transitions (as shown in Figure 3c) was analyzed in order to design the transition. The taper width (*w*) was obtained using the CST optimizer tool with the goal of maximizing the $S_{21}$ parameter at the central frequency of 5.8 GHz. The taper length should be a multiple of a quarter of a guided wavelength [24,25], and the best performance is obtained with tapers that are 3λ/4 long. The taper dimensions of transition C obtained by optimization are listed in Table 4.

**Figure 6:**
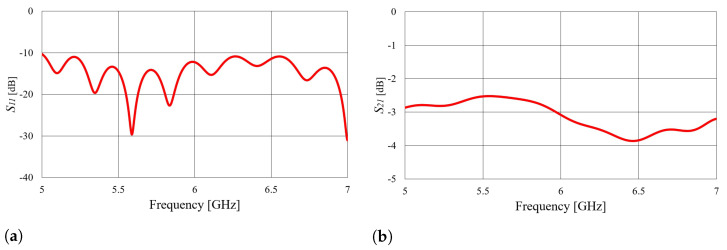
Simulated magnitude of *S* parameters of the two-port cascaded network of transition C (calculated at the SMA ports of the cascaded network): (**a**) $S_{11}$ parameter; (**b**) $S_{21}$ parameter. The considered structure is illustrated in Figure 3c.

Figure 6 shows the simulated $S_{11}$ and $S_{21}$ parameters of a double-cascaded transition network. Based on the *S* parameters shown in Figure 6, it can be concluded that such a transition radiates a significant amount of power. The two-port cascaded network of the two transitions C exhibits a drop in the $S_{21}$ parameter (power transfer) of approximately 3 dB because the dielectric used inside the microstrip and waveguide is foam (with permittivity of $\varepsilon_{r}=1.06$), and the E field does not become trapped below the microstrip and taper lines due to the absence of contrast in permittivity. Using a dielectric with a higher permittivity for transition realization would also imply using the same dielectric as a mold for the textile waveguide antenna. However, a slotted waveguide antenna is, in principle, a leaky wave antenna, and efficient slot radiation requires no permittivity contrast between the waveguide filling and air.

## 4. Experimental Characterization of Textile Slotted Waveguide Antennas

With the purpose of further investigating and verifying the designs proposed in the previous chapter, we fabricated six textile antennas (four for the off-body mode of operation and two for the on-body mode of operation) and experimentally characterized them. All antennas were sewn using the technique presented in [18,19], which involved cutting, folding, and sewing both the slots and edges of the fabric to prevent tearing. A sewing machine was used to sew all seams except the short-circuit terminations, which were sewn by hand. Before sewing the short-circuit terminations, the fabric was pulled onto either a rigid Styrodur or bendable foam mold. The antenna cut was fashioned to ensure a tight fit between the sewn fabric and the mold. Even if a small air gap exists between the fabric and the mold, it has negligible effects on the antenna parameters because the permittivity of the mold is similar to that of air ($\varepsilon_{r}$ = 1.02 or $\varepsilon_{r}$ = 1.06). Finally, the SMA pin was cut to the desired length of transition A, inserted at the correct distance at the bottom of the antenna, and taped to the fabric using conductive tape. We also tested using conductive glue to mount the SMA connectors. The obtained electrical and mechanical properties were excellent, so all the latest designs are made in this way [26,27].

### 4.1. Waveguide Antennas for Off-Body Mode of Operation

**Figure 7:**
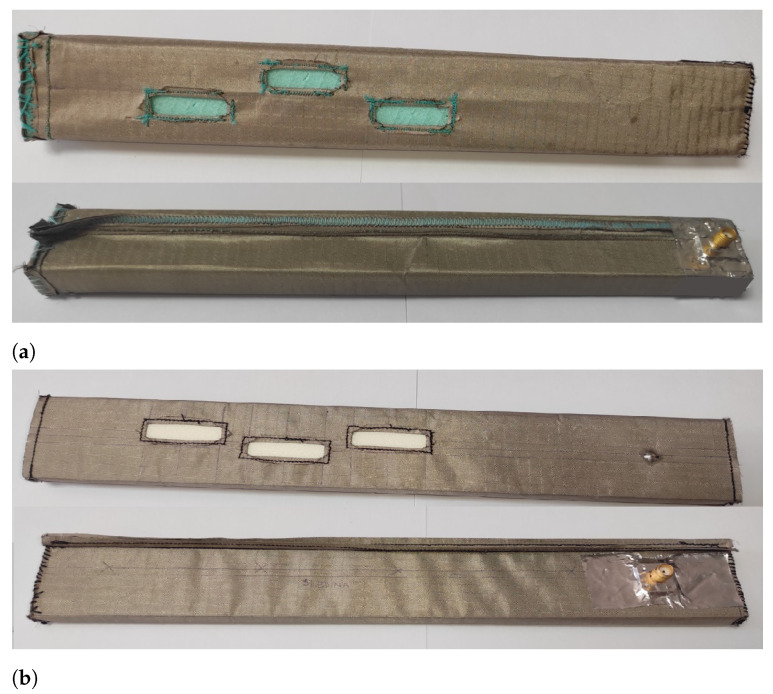
(**a**) Textile antenna with rigid mold; (**b**) textile antenna with bendable mold.

The realized antennas for the off-body mode of operation with a bendable mold with a height of b=6.35 mm and a rigid mold with a height of b=15.8 mm, both with transition A as a feed, are shown in Figure 7. The dimensions of the antennas are listed in Table 5.

**Figure 8:**
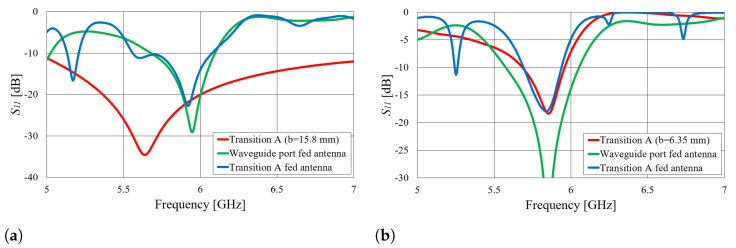
Simulated magnitude of the $S_{11}$ parameter of the considered structures in the two-step design procedure: (**a**) textile waveguide with rigid mold; (**b**) textile waveguide with bendable mold.

**Figure 9:**
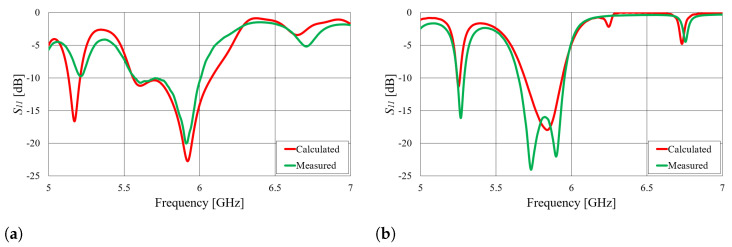
Comparison of the calculated and measured magnitude of the $S_{11}$ parameter of the slotted waveguide antennas: (**a**) textile waveguide with rigid mold; (**b**) textile waveguide with bendable mold.

**Figure 10:**
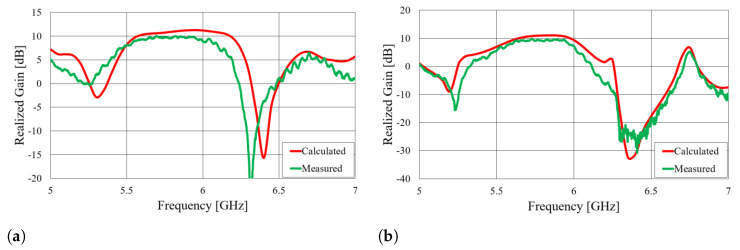
Comparison of calculated and measured realized gain of the slotted waveguide antennas in the broadside direction: (**a**) textile waveguide with rigid mold; (**b**) textile waveguide with bendable mold.

**Figure 11:**
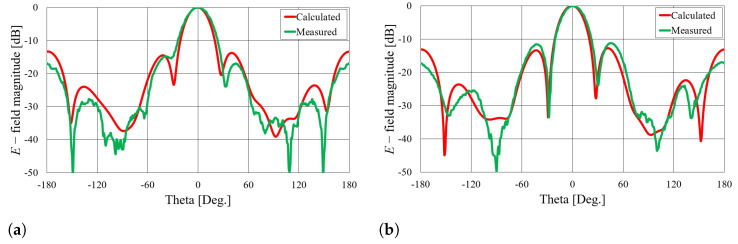
Comparison of calculated and measured radiation pattern of the slotted waveguide antennas in the H plane (i.e., in the yz plane) at 5.8 GHz: (**a**) textile waveguide with rigid mold; (**b**) textile waveguide with bendable mold. The coordinate system is shown in Figure 2a.

**Figure 12:**
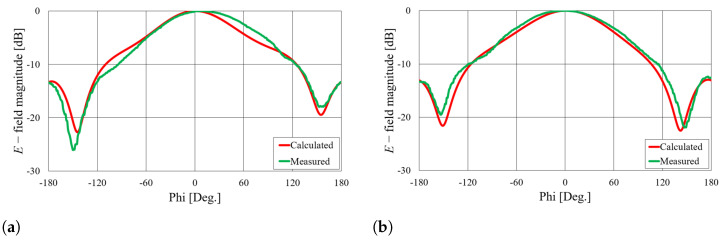
Comparison of calculated and measured radiation pattern of the slotted waveguide antennas in the E plane (i.e., in the xy plane) at 5.8 GHz: (**a**) textile waveguide with rigid mold; (**b**) textile waveguide with bendable mold. The coordinate system is shown in Figure 2a.

First, we verified the two-step design procedure. The calculated $S_{11}$ parameters of the isolated transition A, of the slotted waveguide antenna fed with the waveguide port and of the whole antenna (waveguide array + transitions A) are presented in Figure 8. The $S_{11}$ curve of the whole antenna resembles the less matched curve of two parts: the isolated transition A and the isolated waveguide array. Additionally, Figure 9, Figure 10, Figure 11 and Figure 12 show a comparison of the calculated and measured $S_{11}$ parameters, realized gains, and radiation patterns in the H and E planes of both antennas at 5.8 GHz. The measurements were conducted in the anechoic chamber at the University of Zagreb, employing the standard method for measuring antenna gain and radiation patterns (for this purpose, a Rohde & Schwarz ZVA-40 vector network analyzer and an RFspin QRH20 reference ridge antenna were utilized). All of the measurements demonstrate excellent agreement with the calculated results. The antenna with a bendable mold exhibits a slightly narrower bandwidth than the antenna with a rigid mold, but it still manages to match the entire 5.8 GHz ISM band. This is due to the antenna thickness, resulting in a feed with increased pin length up to the waveguide wall on the opposite side. Furthermore, the slots of the bendable antenna have a shorter slot offset (*s*) in order to reduce the coupling of the slot mode and ensure that the antenna continues to operate in leaky-wave regimes. The realized gains of both antennas are only about 1 dB lower than the calculated values in the 5.8 GHz ISM band of interest, which is an excellent result for textile-type antennas. The measured radiation patterns also agree well with the calculated results.

The results clearly demonstrate that the textile waveguide antenna with a thin flexible mold exhibits comparable microwave performance to the textile antenna with a thick rigid mold. These findings confirm the feasibility of utilizing a thin flexible textile waveguide antenna in various wearable applications. The results also validate the effectiveness of the design procedure for both the transition and antenna array, as well as the fabrication process of the antenna itself.

**Figure 13:**
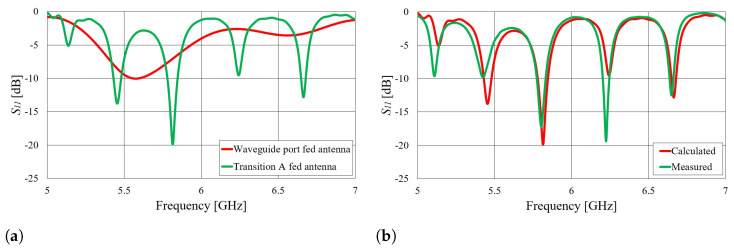
(**a**) Comparison of the simulated magnitude of the $S_{11}$ parameter of a textile waveguide array fed with the waveguide port and transition A; (**b**) comparison of the stimulated and measured $S_{11}$ parameter of the realized alternate textile waveguide array.

**Figure 14:**
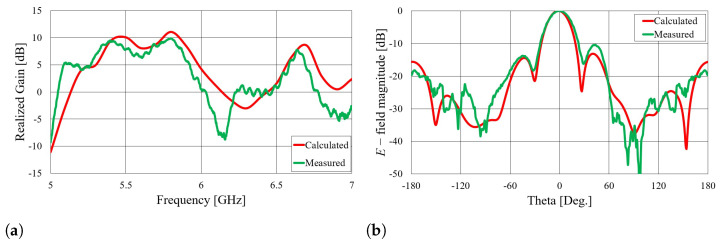
Comparison of calculated and measured realized gain in the broadside direction (**a**) and the radiation pattern in the H plane at 5.8 GHz (**b**) of the alternate antenna design.

Another effect can be observed in the $S_{11}$ parameter, as presented in Figure 8b and Figure 9b. Both the waveguide array fed by the waveguide port and transition A are matched in the 5.8 GHz ISM band, resulting in matching of the whole antenna (slotted waveguide + coax-to-waveguide transition). However, for other frequencies, where transition A or the antenna itself does not provide matching, new modes occur. To further investigate this, the following experiment is presented. Another prototype antenna for the off-body mode of operation was designed for a different frequency band (again, a textile slotted waveguide array with three slots similar to those in Figure 7). The major difference in this case was that the waveguide array excited with the waveguide port was not matched in the 5–7 GHz frequency range. Nevertheless, the whole antenna was still matched in multiple narrow bands when fed with a specially designed transition A, as shown in Figure 13a. In other words, the feed transition was designed to obtain matching of the whole antenna at 5.8 GHz, i.e., the whole antenna was considered in the optimization procedure. In this experiment, the antenna acts as a loaded cavity resonator, and matching is achieved only through the matching of the transition and the length of the resonating antenna (in any case, the slot array is designed to radiate part of both forward and backward propagating waves; see [18]). Approximate modes for such an antenna can then be obtained according to Equation (1).

(1) $$f_{m,n,p}=\frac{c_{0}}{2\sqrt{\varepsilon_{r}}}\sqrt{{\left(\frac{m}{a}\right)}^{2}+{\left(\frac{n}{b}\right)}^{2}+{\left(\frac{p}{c}\right)}^{2}},$$

where $c_{0}$ represents the speed of light, and *a*, *b*, and *c* are the dimensions of the rectangular waveguide (resonator). $\varepsilon_{r}$ denotes the relative permittivity of the mold, and *m*, *n*, and *p* indicate the number of space modes in each direction. However, it is important to note that the narrow operating bandwidth of the antenna is a tradeoff in achieving matching through the transition, as opposed to the previous antenna, which was somewhat broadly matched. The measured $S_{11}$ parameter, realized gain, and radiation pattern in the H plane at 5.8 GHz are presented in Figure 13 and Figure 14. All the results are in agreement with the calculated results.

**Figure 15:**
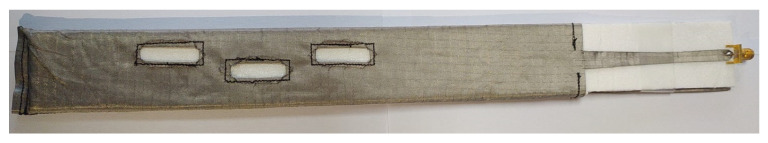
Textile waveguide antenna fed with transition C.

**Figure 16:**
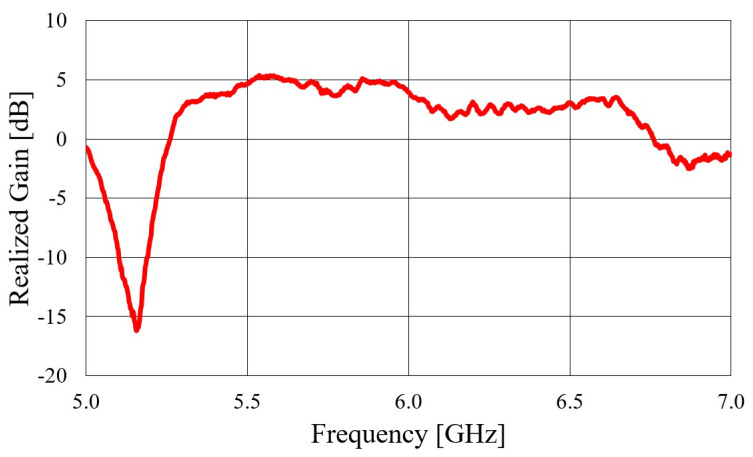
Measured realized gain in the broadside direction of the textile waveguide antenna fed with transition C.

Next, a textile microstrip off-body antenna fed with transition C was designed. The fabricated antenna is shown in Figure 15, and its measured realized gain is presented in Figure 16. From the realized gain plot, it is evident that the antenna fed with transition C performs significantly worse than the prior textile waveguide antennas. This poor performance is primarily due to the microstrip transition and the substrate used, which has a low contrast in permittivities (ε=1.06). The low contrast causes the EM fields not to be concentrated under the microstrip conductor, i.e., to leak out of the substrate, leading to significant energy loss. Therefore, the design of the presented antenna fed with transition C is not a viable solution.

### 4.2. Waveguide Antennas for On-Body Mode of Operation

**Figure 17:**
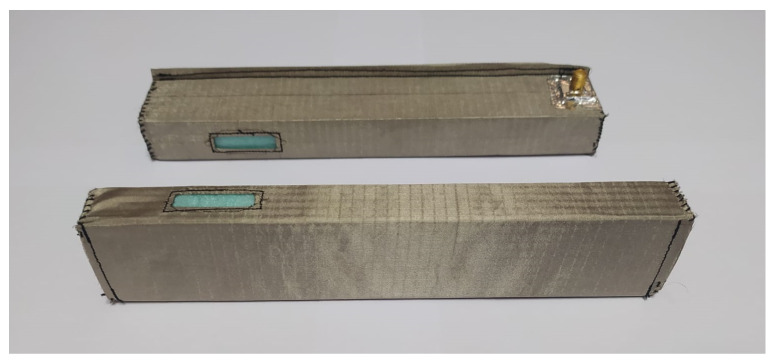
On-body textile waveguide antennas fed with transition A.

Two identical on-body textile waveguide antennas with rigid molds were designed, integrated with transition A, and experimentally characterized. To enable the launch of the normally polarized EM wave, slots were cut in the narrow walls of the waveguide. The rigid molds used for these antennas have slightly different cross-sectional dimensions compared to the rigid mold used for the off-body antenna. As a result, the dimensions of transition A were modified and optimized accordingly. The optimized dimensions of transition A, along with the dimensions of the on-body antennas, are presented in Table 6. The two realized on-body antennas are shown in Figure 17.

**Figure 18:**
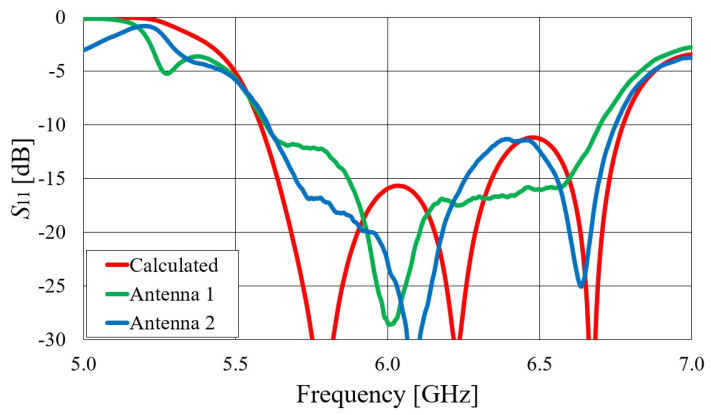
Comparison of calculated and measured $S_{11}$ parameters of on-body textile waveguide antennas.

**Figure 19:**
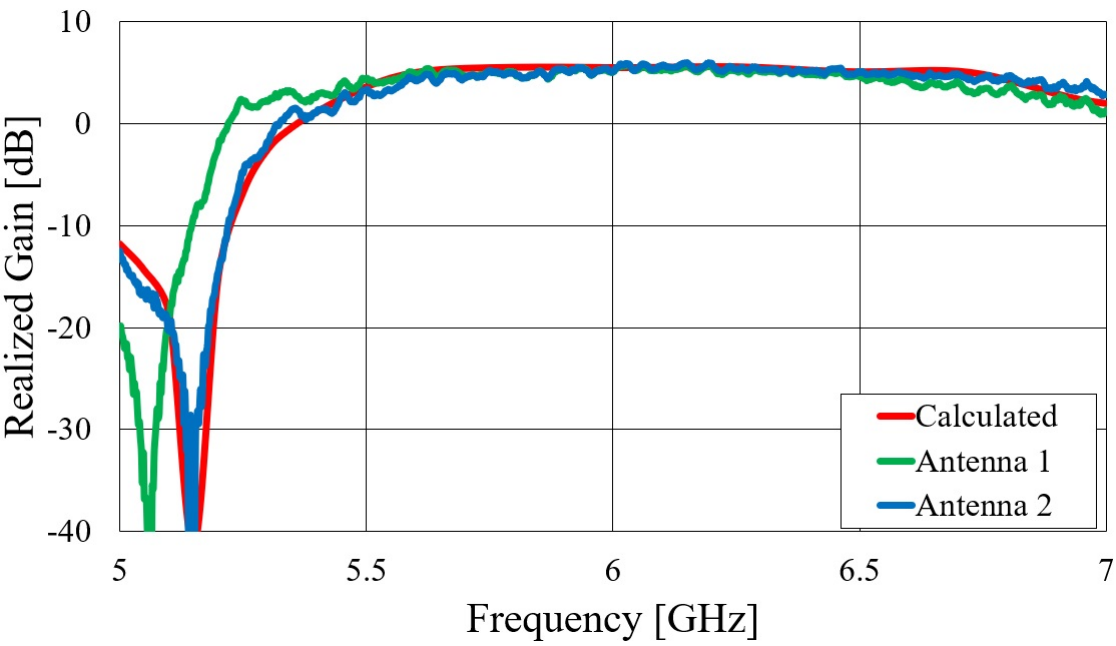
Comparison of calculated and measured realized gain in the direction of maximum radiation of on-body textile waveguide antennas.

**Figure 20:**
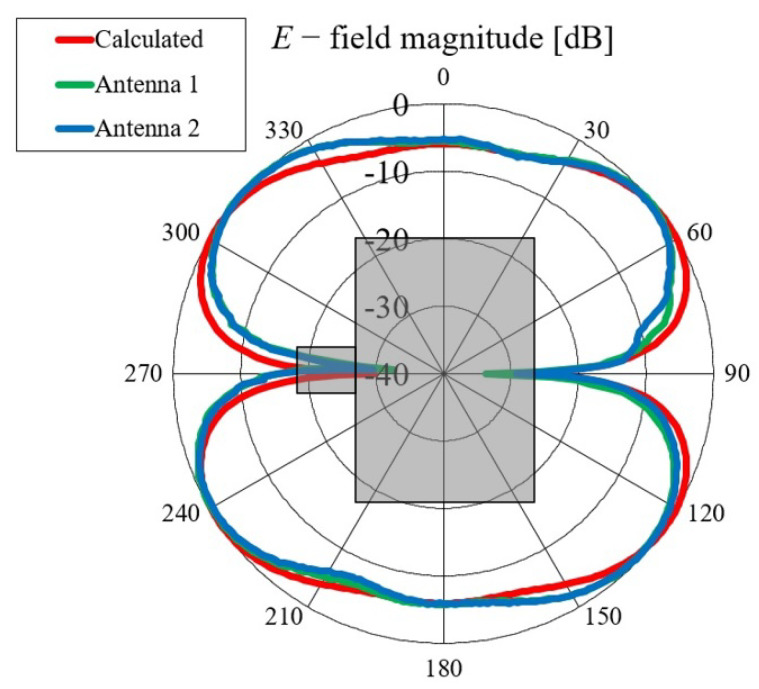
Comparison of calculated and measured radiation patterns in the *E* plane at 5.8 GHz.

**Figure 21:**
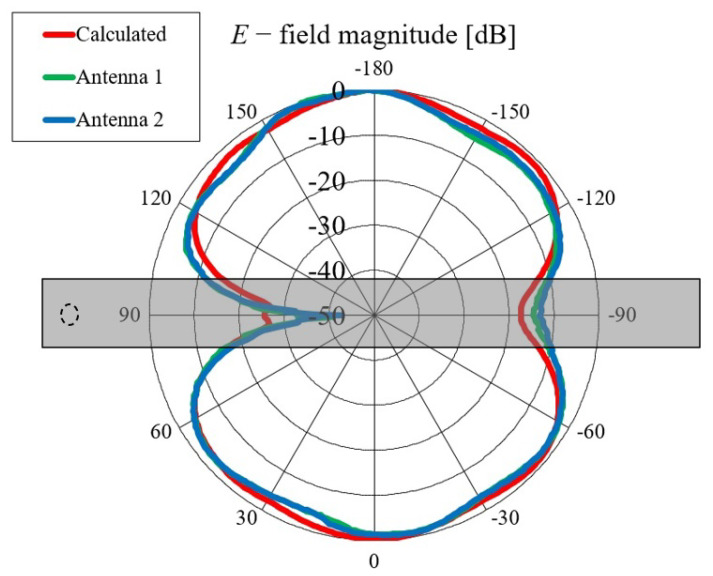
Comparison of calculated and measured radiation patterns in the *H* plane at 5.8 GHz.

Comparisons of the calculated and measured $S_{11}$ parameters, realized gains in the direction of maximum radiation, and radiation patterns in the *E* and *H* planes at 5.8 GHz for both on-body antennas are presented in Figure 18, Figure 19, Figure 20 and Figure 21. Both antennas are broadly matched in the 5.6–6.7 GHz band. The realized gain for both antennas is very stable in the band of interest and remarkably close to the calculated values, thereby demonstrating the quality of the design and sewing process. The radiation patterns in both planes also agree with the calculated results. Figure 21 reveals that the antenna launches the EM waves along the body in a wide angular range so that communication between the access point and the sensor units along the body is possible in all directions except maybe in the direction along the waveguide axis. The obtained results also demonstrate that the manufacturing process of the considered textile waveguide antennas is repeatable, i.e., there are small differences in the measured parameters of the two realized antennas.

**Figure 22:**
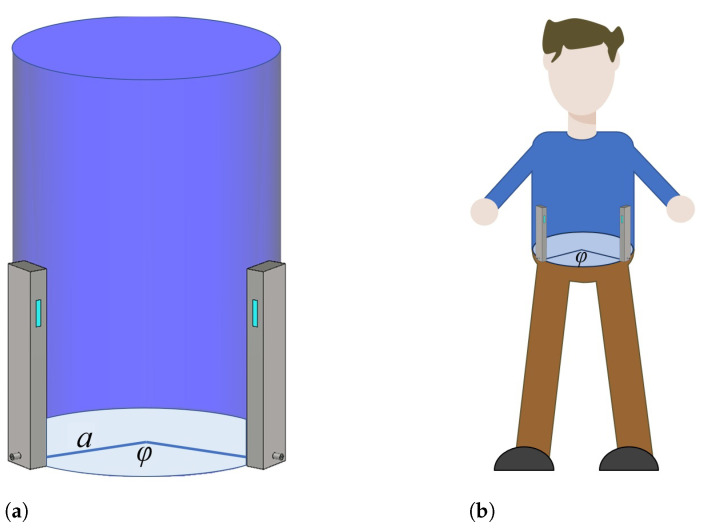
Measurement of the $S_{21}$ parameter between wearable antennas placed on a body phantom (**a**) and on a human body (**b**).

To verify the feasibility of the on-body antenna design, the fabricated antennas were tested and measured in the presence of the human body. Two on-body antennas were placed vertically on a cylindrical phantom (with dimensions of a=12.5 cm, height h=30 cm) filled with liquid representing the electromagnetic properties of the human body at 5.8 GHz, and their positions were varied along the rim of the phantom. In other words, according to the measurement setup illustrated in Figure 22a, the axes of the waveguide antennas and the axis of the cylindrical phantom were parallel to each other, and the antennas were attached to the phantom with the wide waveguide wall and spaced in the circumferential direction (the angular distance (φ) between the antennas refers to the distance between the axes of the waveguide antennas). The liquid used was a 20% ABV ethanol solution ($\varepsilon_{r}=50.89,\sigma =8.64$ S/m [28]). Note that the $S_{21}$ parameter of the two-antenna system represents the power transfer between antennas. In addition, the antennas were placed on the human body, and the $S_{21}$ parameter was measured, as shown in Figure 22b (the measurement setup is identical to that with a cylindrical phantom). The human body was modeled in CST using the IEEE body model in the 5.8 GHz ISM band (ε=48.2,σ=6 S/m [29]), and the human shape was approximated as a cylinder with an elliptical cross section with the length of the major and minor axes equal 35 cm and 25 cm, respectively.

**Figure 23:**
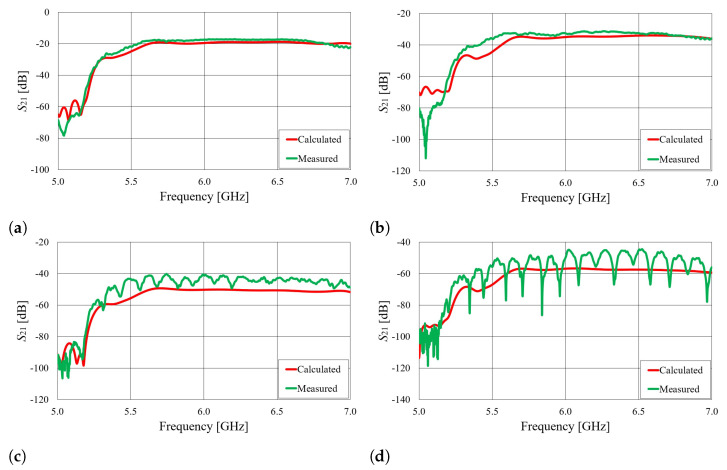
The $S_{21}$ parameter of on-body antennas with angular separation of $\varphi =45^{\circ }$ (**a**), $\varphi =90^{\circ }$ (**b**), $\varphi =135^{\circ }$ (**c**), and $\varphi =180^{\circ }$ (**d**). The antennas were placed on a cylindrical phantom filled with 20% ABV ethanol.

**Figure 24:**
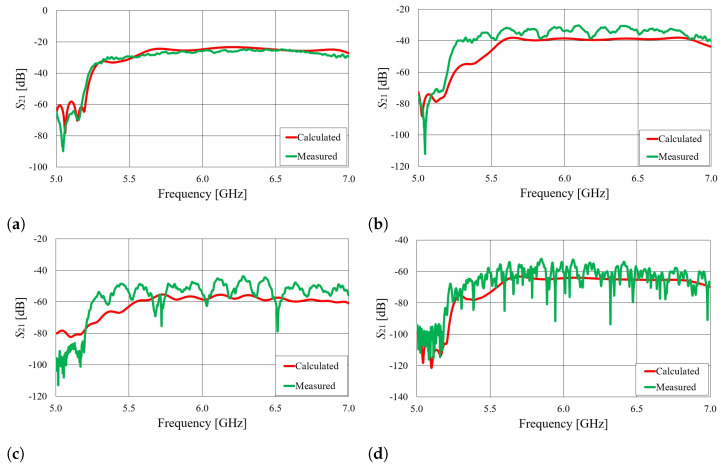
The $S_{21}$ parameter of on-body antennas with angular separation of $\varphi =45^{\circ }$ (**a**), $\varphi =90^{\circ }$ (**b**), $\varphi =135^{\circ }$ (**c**), and $\varphi =180^{\circ }$ (**d**). The antennas were placed on the human body.

The $S_{21}$ parameters of the on-body wearable antennas placed on the cylindrical phantom filled with 20% ABV ethanol, as well as for the real human body, are shown in Figure 23 and Figure 24. It can be observed that the measured signal values closely match the calculated results.

**Figure 25:**
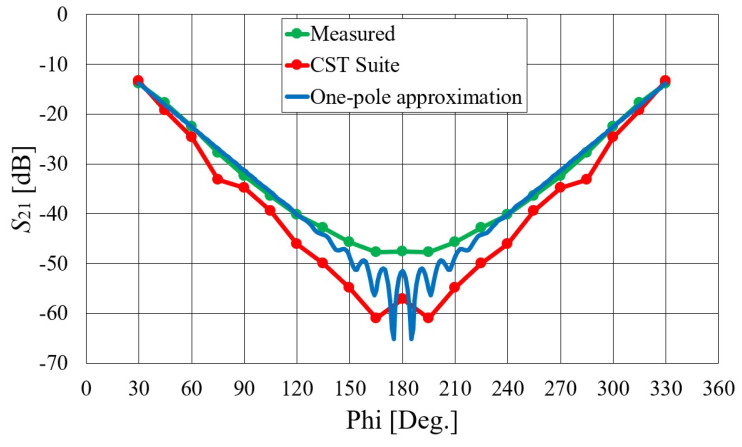
The $S_{21}$ parameter of on-body antennas at 5.8 GHz with respect to the angular distance between antennas. The antennas were placed on a cylindrical phantom filled with 20% ABV ethanol.

Figure 25 illustrates the signal attenuation with respect to the angular distance between antennas in the 5.8 GHz ISM band. The graph represents an ethanol-filled circular–cylindrical phantom. The displayed measurement data are average signal values in the 5.7–6.5 GHz frequency range (as shown in Figure 23, both the theoretical and experimental $S_{21}$ characteristics are almost constant in that frequency band).

For practical reasons, it would be useful to determine the approximate dependence of the $S_{21}$ parameter on the angular distance between the antennas, i.e., to estimate the coupling level in the on-body mode of operation. This can be done by expressing the magnetic field (i.e., the H-field Green’s function due to axially oriented magnetic source) as a sum of creeping wave modes [30,31]:

(2) $$H_{z}\left(\rho ,\phi \right)=2\pi j\sum_{k}A_{k}\left(\rho \right)\left(e^{-j\nu_{k}\phi }+e^{-j\nu_{k}\left(2\pi -\phi \right)}\right),$$

where the two terms in brackets represent EM waves traveling clockwise and counterclockwise around the cylinder. The complex poles ($\nu_{k}$) are determined as poles of the considered spectral-domain Green’s function in the ν-complex plane [32]. In our case, they are determined by solving the following characteristic equation:

(3) $$\sqrt{{\bar{\epsilon }}_{r}}J_{\nu_{k}}\left(k_{0}\sqrt{{\bar{\epsilon }}_{r}}a\right){H_{\nu_{k}}^{\left(2\right)}}^{\prime }\left(k_{0}a\right)-{J_{\nu_{k}}}^{\prime }\left(k_{0}\sqrt{{\bar{\epsilon }}_{r}}a\right)H_{\nu_{k}}^{\left(2\right)}\left(k_{0}a\right)=0$$

where $k_{0}=\omega /c_{0}$ is the free-space propagation constant; ${\bar{\epsilon }}_{r}$ is the complex relative permittivity (${\bar{\epsilon }}_{r}=\epsilon_{r}-j\sigma /\omega \epsilon_{0}$); *a* is the radius of the cylindrical phantom; and $J_{\nu_{k}}$ and $H_{\nu_{k}}^{\left(2\right)}$ are the $\nu_{k}$-order Bessel and Hankel functions of the second kind, respectively. They are calculated using Olver’s expansion as described in [33], while the algorithm for finding complex roots of the characteristic Equation (3) is presented in [34]. The complex zeros ($\nu_{k}$) can be interpreted as the angular propagation coefficients of two EM waves traveling around the cylinder in opposite directions. The imaginary parts of $\nu_{k}$ represent the losses arising from creeping of the EM energy into space, so the field magnitude of each considered wave is exponentially attenuated when propagating around the body. The approximated propagation and attenuation constants are related to the dominant creeping mode, and in the considered case, they are equal to $\nu_{1}/k_{0}a=1.092-j0.127$. The coupling results obtained with the dominant creeping wave approach are also presented in Figure 25. There is a strong interference pattern for separation angles around $180^{\circ }$, since two creeping waves that propagate around the cylindrical phantom in opposite directions (Equation (2)) are of the same order of magnitude in that angular range, resulting in constructive and destructive interference. These ripples are not visible in the measured results because we expressed them as an average over the frequency band.

## 5. Conclusions

The presented work demonstrates the feasibility of using textile waveguide antennas for wearable applications. In this study, we considered different feeding structures, characterized them through simulation and experiment, and identified the most suitable feeding structure for the considered waveguide antenna. The selection criteria for the feeding structure were based on electrical performance, simplicity of construction, and ease of mounting on the waveguide antenna. The achieved frequency bandwidth was mainly determined by the number of degrees of freedom in the feed design procedure. In addition, it was demonstrated that foam-based molds and related thin flexible textile waveguide antennas show no performance degradation compared to thick rigid antennas. The fabricated antennas exhibited excellent electromagnetic properties, proving the proposed concepts. Two body-centric wireless scenarios were tested: off-body and on-body modes of operation. For the latter, a simple approximate formula was examined that accurately predicts the coupling level between wearable antennas. The presented work provides a basis for further development and optimization of wearable slotted waveguide antennas for various applications, such as smart uniforms, safety vests, and body sensor networks. 

## Figures and Tables

**Table 1:** Waveguide dimensions.

	Antenna with Rigid Mold	Antenna with Bendable Mold
Waveguide width (*a*)	34.0 mm	35.0 mm
Waveguide height (*b*)	15.8 mm	6.35 mm
Mold permittivity ($\varepsilon_{r}$)	1.02	1.06

**Table 2:** Parameters of transition A (dimensions according to Figure 3a).

	Antenna with Rigid Mold	Antenna with Bendable Mold
Pin length ($H_{p}$)	10.0 mm	6.35 mm
Distance from		
short-circuit wall ($L_{sc}$)	9.0 mm	30.2 mm

**Table 3:** Parameters of transition B (dimensions according to Figure 3b).

	Antenna with Rigid Mold		Antenna with Bendable Mold	
Pin/strip				
length	$L_{w}=15.63$ mm	$L_{s}=15.0$ mm	$L_{w}=22.13$ mm	$L_{s}=21.0$ mm
Pin/strip				
diameter/width	$D_{w}=1.3$ mm	$W_{s}=4.0$ mm	$D_{w}=1.3$ mm	$W_{s}=4.0$ mm
Vertical				
wire length	$H_{p}=7.9$ mm	$H_{p}=7.9$ mm	$H_{p}=13.8$ mm	$H_{p}=13.8$ mm
Vertical				
wire diameter	$D_{p}=0.5$ mm	$D_{p}=1.0$ mm	$H_{p}=0.5$ mm	$H_{p}=1.0$ mm

**Table 4:** Dimensions of transition C.

50 Ω microstrip line width ($w_{0}$)	4.46 mm
Taper width (*w*)	7.22 mm
50 Ω microstrip line length (*s*)	30 mm
Taper length (*l*)	52 mm
Waveguide width (*a*)	34.85 mm
Waveguide height (*b*)	6.35 mm
50 Ω microstrip line height (*h*)	1.5 mm

**Table 5:** Textile waveguide off-body antenna dimensions.

	Antenna with Rigid Mold	Antenna with Bendable Mold
Slot length (*l*)	24.5 mm	26.4 mm
Slot width (*w*)	6.0 mm	6.0 mm
Slot offset (*s*)	5.5 mm	3.2 mm
Slot spacing (*d*)	38.0 mm	36.5 mm
Spacing from SC ($d_{sc}$)	57.0 mm	54.8 mm

**Table 6:** Textile waveguide on-body antennas with rigid mold dimensions (according to Figure 2b and Figure 3a).

Waveguide width (*a*)	37.5 mm
Waveguide height (*b*)	19.5 mm
Antenna length (*L*)	200 mm
Pin length ($H_{p}$)	10.6 mm
Distance from short-circuit wall ($L_{sc}$)	9.5 mm
Slot length (*l*)	26.7 mm
Slot width (*w*)	6.0 mm
Spacing from SC ($d_{sc}$)	43.3 mm

## References

[1] Jara A.J. Wearable Internet: Powering Personal Devices with the Internet of Things Capabilities. Proceedings of the 2014 International Conference on Identification, Information and Knowledge in the Internet of Things.

[2] Paracha K.N., Rahim S.K.A., Soh P.J., Khalily M. (2019). Wearable Antennas: A Review of Materials, Structures, and Innovative Features for Autonomous Communication and Sensing. IEEE Access.

[3] Whittow W. (2022). Bioelectromagnetics in Healthcare: Advanced Sensing and Communication Applications.

[4] Hall P.S., Hao Y. (2012). Antennas and Propagation for Body-Centric Wireless Communications.

[5] Babic J., Bilic M., Kovac I. Safety Vest System for Human-Robot Collaboration. Proceedings of the 2022 International Convention on Information, Communication and Electronic Technology (MIPRO).

[6] Corchia L., Monti G., Tarricone L. (2019). Wearable antennas: Nontextile versus fully textile solutions. IEEE Antennas Propag. Mag..

[7] Roh J.S., Chi Y.S., Kang T.J. (2010). Wearable textile antennas. Int. J. Fash. Des. Technol. Educ..

[8] Mahmood S.N., Ishak A.J., Saeidi T., Alsariera H., Alani S., Ismail A., Soh A. (2020). Recent Advances in Wearable Antenna Technologies: A Review. Prog. Electromagn. Res. B.

[9] Ivsic B., Bonefacic D., Bartolic J. (2012). Considerations on Embroidered Textile Antennas for Wearable Applications. IEEE Antennas Wirel. Propag. Lett..

[10] Nepa P., Rogier H. (2015). Wearable Antennas for Off-Body Radio Links at VHF and UHF Bands: Challenges, the state of the art, and future trends below 1 GHz. IEEE Antennas Propag. Mag..

[11] Hirokawa J., Zhang M., Chen Z. (2016). Waveguide Slot Array Antennas. Handbook of Antenna Technologies.

[12] Sanz-Izquierdo G.B., Wu L., Batchelor J.C., Young P.R. Textile integrated waveguide slot antenna. Proceedings of the 2010 IEEE Antennas and Propagation Society International Symposium.

[13] Lemey H.S., Rogier H. Substrate integrated waveguide textile antennas as energy harvesting platforms. Proceedings of the 2015 International Workshop on Antenna Technology (iWAT).

[14] Alonso L., Ver Hoeye S., Fernández M., Vazquez C., Camblor R., Hotopan G., Hadarig A., Las-Heras F. Millimetre wave textile integrated waveguide beamforming antenna for radar applications. Proceedings of the Global Symposium on Millimeter-Waves.

[15] Lajevardi M.E., Kamyab M. (2017). Ultraminiaturized metamaterial-inspired SIW textile antenna for off-body applications. IEEE Antennas Wirel. Propag. Lett..

[16] Kokolia M., Raida Z. AMC-Based Textile-Integrated Antenna. Proceedings of the 23rd International Microwave and Radar Conference (MIKON).

[17] Kokolia D., Raida Z. (2021). Textile-integrated microwave components based on artificial magnetic conductor. Int. J. Numer. Model..

[18] Mikulic D., Sopp E., Bonefacic D., Sipus Z. (2022). Textile slotted waveguide antennas for body-centric applications. Sensors.

[19] Mikulic D., Sopp E., Bonefacic D., Sipus Z. Wearable Slotted Waveguide Textile Antenna. Proceedings of the 16th European Conference on Antennas and Propagation (EuCAP).

[20] Mikulic D., Sopp E., Bonefacic D., Sipus Z. Textile Slotted Waveguide Antenna: Feeding Considerations. Proceedings of the 17th European Conference on Antennas and Propagation (EuCAP).

[21] Shieldex. https://www.shieldex.de/en/products/shieldex-nora-dell/.

[22] Cuming Microwave C-FOAM PF-2 and PF-4. https://www.cumingmicrowave.com/dielectric-materials-application/c-foam-pf-2-and-pf-4.html.

[23] CST Studio Suite Dassault Systèmes. https://www.3ds.com/products-services/simulia/products/cst-studio-suite/.

[24] Kumar H., Jadhav R., Ranade S. (2012). A Review on Substrate Integrated Waveguide and its Microstrip Interconnect. J. Electron. Commun. Eng..

[25] Deslandes D. Design equations for tapered microstrip-to-Substrate Integrated Waveguide transitions. Proceedings of the IEEE MTT-S International Microwave Symposium Digest.

[26] Bonefacic D. Conductive Adhesives at Microwave Frequencies: Silver-Filled vs. Graphite-Filled. Proceedings of the 17th European Conference on Antennas and Propagation (EuCAP).

[27] Hollandshielding. https://hollandshielding.com/Electrically-conductive-adhesive-shieldokit.

[28] Song H., Wei B., Yu Q., Xiao X., Kikkawa T. (2020). WiEps: Measurement of Dielectric Property With Commodity WiFi Device—An Application to Ethanol/Water Mixture. IEEE Internet Things J..

[29] Federal Communications Commission (FCC) (2001). Evaluating Compliance with FCC Guidelines for Human Exposure to Radiofrequency Electromagnetic Fields.

[30] Wait J.R. (1959). Electromagnetic Radiation From Cylindrical Structures.

[31] Alves T., Poussot B., Laheurte L.-M. (2011). Analytical Propagation Modeling of BAN Channels Based on the Creeping-Wave Theory. IEEE Trans. Antennas Propag..

[32] Ivsic B., Bonefacic D., Sipus Z., Bartolic J. (2017). An Insight into Creeping Electromagnetic Waves around the Human Body. Wirel. Commun. Mob. Comput..

[33] Paknys R. (1992). Evaluation of Hankel Functions with Complex Argument and Complex Order. IEEE Trans. Antennas Propag..

[34] Galdi V., Pinto I.M. (2000). A simple algorithm for accurate location of leaky-wave poles for grounded inhomogeneous dielectric slabs. Microwave Opt. Technol. Lett..

